# Measurement of apolipoprotein E and amyloid β clearance rates in the mouse brain using bolus stable isotope labeling

**DOI:** 10.1186/1750-1326-7-14

**Published:** 2012-04-18

**Authors:** Jacob M Basak, Jungsu Kim, Yuriy Pyatkivskyy, Kristin R Wildsmith, Hong Jiang, Maia Parsadanian, Bruce W Patterson, Randall J Bateman, David M Holtzman

**Affiliations:** 1 Department of Neurology, Saint Louis, Missouri, 63110, USA; 2 Medicine, Saint Louis, Missouri, 63110, USA; 3 Developmental Biology, Saint Louis, Missouri, 63110, USA; 4 Hope Center for Neurological Disorders, Saint Louis, Missouri, 63110, USA; 5 Knight Alzheimer’s Disease Research Center, Washington University School of Medicine, Saint Louis, Missouri, 63110, USA

**Keywords:** Stable isotope, Apolipoprotein E, Amyloid beta, Kinetics, Protein turnover, LDLR, ABCA1, Multiple reaction monitoring mass spectrometry

## Abstract

**Background:**

Abnormal proteostasis due to alterations in protein turnover has been postulated to play a central role in several neurodegenerative diseases. Therefore, the development of techniques to quantify protein turnover in the brain is critical for understanding the pathogenic mechanisms of these diseases. We have developed a bolus stable isotope-labeling kinetics (SILK) technique coupled with multiple reaction monitoring mass spectrometry to measure the clearance of proteins in the mouse brain.

**Results:**

Cohorts of mice were pulse labeled with ^13^ C_6_-leucine and the brains were isolated after pre-determined time points. The extent of label incorporation was measured over time using mass spectrometry to measure the ratio of labeled to unlabeled apolipoprotein E (apoE) and amyloid β (Aβ). The fractional clearance rate (FCR) was then calculated by analyzing the time course of disappearance for the labeled protein species. To validate the technique, apoE clearance was measured in mice that overexpress the low-density lipoprotein receptor (LDLR). The FCR in these mice was 2.7-fold faster than wild-type mice. To demonstrate the potential of this technique for understanding the pathogenesis of neurodegenerative disease, we applied our SILK technique to determine the effect of ATP binding cassette A1 (ABCA1) on both apoE and Aβ clearance. ABCA1 had previously been shown to regulate both the amount of apoE in the brain, along with the extent of Aβ deposition, and represents a potential molecular target for lowering brain amyloid levels in Alzheimer's disease patients. The FCR of apoE was increased by 1.9- and 1.5-fold in mice that either lacked or overexpressed ABCA1, respectively. However, ABCA1 had no effect on the FCR of Aβ, suggesting that ABCA1 does not regulate Aβ metabolism in the brain.

**Conclusions:**

Our SILK strategy represents a straightforward, cost-effective, and efficient method to measure the clearance of proteins in the mouse brain. We expect that this technique will be applicable to the study of protein dynamics in the pathogenesis of several neurodegenerative diseases, and could aid in the evaluation of novel therapeutic agents.

## Background

In the proteomics era, significant effort has been devoted to developing techniques that accurately and efficiently determine differences in protein amounts under normal physiological conditions and disease states [1]. However, quantifying protein turnover rates at both a cellular and systemic level is also necessary for a complete understanding of the mechanisms dictating changes in protein levels [2]. Several neurodegenerative diseases are characterized by the accumulation of protein aggregates in the brain, including Alzheimer’s disease (AD) [3], Parkinson’s disease [4], Huntington’s disease [5], and frontotemporal dementia[6]. Although the underlying cause of protein aggregation in these diseases remains unclear, it is likely due to abnormal proteostasis caused by alterations in protein production or clearance [7,8]. Therefore, the development of techniques that can assess protein dynamics in the brain are fundamental for advancing our understanding of these disease processes and aiding the conception of innovative therapeutics.

Stable isotope tracers have been in use for many years to facilitate the analysis of protein turnover in cells and whole organisms [9]. Mass spectrometry (MS) has proven an effective tool for the analysis of stable isotope incorporation into individual proteins [10]. Liquid chromatography-mass spectrometry (LC-MS) analysis allows for the comparison of the relative abundance of labeled to unlabeled peptides due to their mass separation. Coupling stable isotope amino acid labeling with LC-MS has been applied to quantify protein synthesis and degradation in yeast [11], mammalian cell lines [12,13], and small animals [14,15]. However, protein turnover studies in animals have been limited due to issues with MS detection sensitivity and accurate label quantification, along with difficulties in achieving cost-effective and practical methods for tracer administration. Recently, Bateman *et al.* have developed a method to measure the dynamics of low abundance proteins in the cerebral spinal fluid (CSF) of humans [16]. In this technique, ^13^ C_6_-leucine is injected intravenously into research participants and samples of the lumbar CSF are serially collected over a predetermined time period. The synthesis and clearance rates of proteins are then measured by quantifying the appearance and disappearance of the ^13^ C_6_-leucine in proteins over time via LC-MS [16,17]. The value of this technique has specifically been highlighted for the amyloid β (Aβ) peptide, which accumulates in the brains of AD patients and has been implicated in the disease pathogenesis [3]. Application of stable isotope labeling to studies of Aβ dynamics have demonstrated impaired Aβ clearance in individuals with AD and the ability of a gamma secretase inhibitor to decrease Aβ synthesis in the CNS [8,16,18].

Apolipoprotein E (apoE) plays a central role in the transport of cholesterol by functioning as a ligand for the receptor-mediated endocytosis of lipoprotein particles into cells [19]. In humans, three common apoE isoforms exist (apoE2, apoE3, and apoE4) that differ by amino acids at positions 112 and 158. ApoE4 is currently the strongest known genetic risk factor for late-onset AD, and as a result significant effort has been devoted to understanding apoE’s physiological function in the brain along with its role in AD pathogenesis [20]. A major hypothesis for how apoE4 affects the onset of AD contends that apoE promotes the aggregation of Aβ into amyloid plaques in the brain, either through impairing Aβ clearance [21,22], directly regulating the propensity of Aβ to form amyloid fibrils [23,24], or both mechanisms. Independent of apoE isoform, the amount of apoE in the brain appears to be critical for determining the extent of amyloid deposition [25,26]. Therefore, finding proteins and molecular pathways that regulate apoE levels in the brain has been the focus of significant attention in the AD research community.

Increasing the endocytosis of apoE via the low-density lipoprotein receptor (LDLR) has been shown to decrease apoE levels in the mouse brain, likely through increased apoE clearance [27]. Brain levels of the cholesterol transporter ATP binding cassette A1 (ABCA1) also alter the amount of apoE and the extent of apoE lipidation in mice. Both the overexpression and deletion of ABCA1 in the mouse brain resulted in a decrease in apoE protein level [28-30]. In transgenic mice that overexpress the human amyloid precursor protein (APP), increasing ABCA1 levels caused a significant decrease in amyloid deposition in the brain [30]. Therefore, it has been hypothesized that ABCA1 regulates amyloid levels in the brain by altering Aβ clearance, and this could occur through the effect of ABCA1 on either apoE lipidation or total apoE levels. However, no study has yet addressed this hypothesis formally by studying apoE and Aβ metabolism in the brain of mice with altered ABCA1 levels.

Herein, we describe a novel method to study protein clearance in the mouse brain. We show that following a bolus injection of ^13^ C_6_-leucine into mice, LC-MS analysis of brain tissue can be used to measure the fractional clearance rate (FCR) of individual proteins. We validate our technique by analyzing changes in the clearance of apolipoprotein E (apoE) in mice that have been genetically engineered to overexpress LDLR. Finally, we use ^13^ C_6_-leucine labeling coupled to LC-MS analysis to measure the clearance of apoE and Aβ in APP transgenic mice that either overexpress or lack ABCA1. Both overexpression and deletion of ABCA1 resulted in an increased fractional clearance rate of apoE. However, ABCA1 levels did not alter the clearance rate of Aβ in the mouse brain, suggesting ABCA1 acts via another pathway, such as directly influencing Aβ aggregation, to regulate amyloid deposition. These results highlight the power of our stable isotope labeling technique in elucidating mechanisms of protein clearance from the brain, and suggest that future studies could use this technique to study the clearance pathways of other proteins implicated in neurodegenerative disease.

## Results and discussion

### Stable isotope labeling of mice and protein isolation

Several methods have been described to study protein turnover in animals using stable isotope labeling. Traditionally, stable isotope incorporation was measured by gas chromatography mass spectrometry (GC-MS) quantitation of labeled amino acids obtained following protein derivatization [9]. However, this technique is limited to measuring large quantities of proteins, and consequently has only been used to analyze total tissue protein turnover or the kinetics of highly abundant proteins [31-33]. Recently the more sensitive analytical technique of LC-MS has been applied to quantify the turnover of specific proteins following administration of a stable amino acid in the diet of animals [14,15]. Though these techniques have provided useful information on the turnover of abundant proteins in various organs, the requirement of labeled amino acid delivery via the diet has been a technical issue. Creating a diet enriched with an isotopic label is costly, and inability to control feeding patterns in animals such as mice and rats requires long term exposure (several hours to days) to the labeled diet to achieve reliable and consistent isotope levels in tissues. This is especially problematic in studying proteins with rapid turnover rates, as the difficulty in accurately measuring label incorporation over short time periods (minutes to hours) limits the sensitivity of kinetic analysis. To create a more practical and efficient method of labeling proteins, we have tested whether pulse labeling of mice could be used to measure protein turnover rates in the mouse brain.

**Figure 1:**
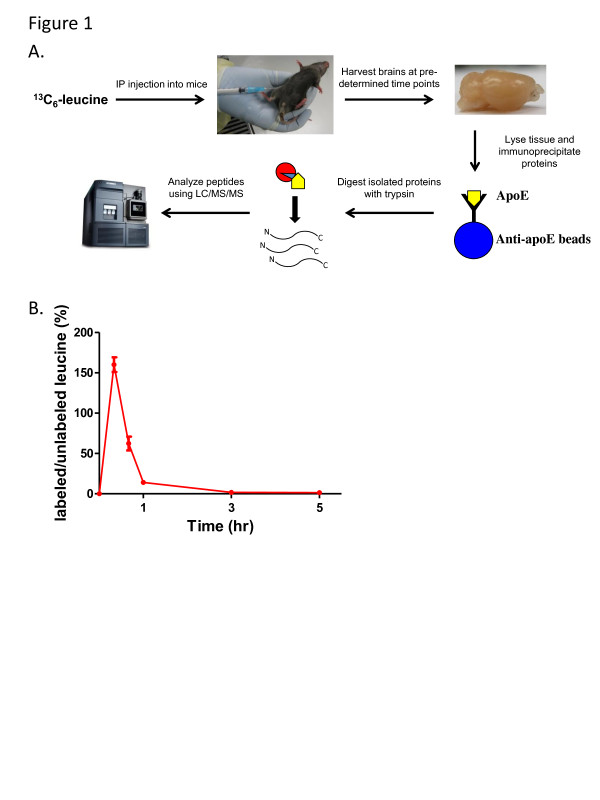
**Experimental schematic for stable isotope labeling and isolation of mouse brain proteins. (A)** Cohorts of mice were pulse-labeled with ^13^ C_6_-leucine via a bolus intraperitoneal injection (200 mg/kg of body weight). After a pre-determined time following the ^13^ C_6_-leucine administration, the mice were euthanized and the brains were removed. The brain tissue was then lysed using a 1% Triton X-100 lysis buffer, and the protein of interest was immunoprecipitated from the brain lysate (apoE is shown as an example). The precipitated proteins were then eluted from the antibody beads and subjected to trypsin digestion. The resulting peptide mixture was separated and analyzed via ultra performance liquid chromatography tandem mass spectrometry (UPLC/MS/MS) (yellow = apoE, blue = sepharose bead, red = trypsin). **(B)** To observe the bioavailability of the ^13^ C_6_-leucine, plasma samples were collected at sequential time points following the bolus injection and subjected to GC-MS analysis. The tracer-to-tracee ratio (TTR, shown as labeled/unlabeled leucine) was then measured by quantifying the relative amounts of ^13^ C_6_-leucine and dividing by the amount of unlabeled leucine in each sample. Each time point in the graph represents the average value from 5–6 individual mice

The outline of our experimental design for the labeling of mice and tissue processing is shown in Figure 1A. Mice were intraperitoneally (IP) injected with a bolus of ^13^ C_6_-leucine, a non-radioactive stable isotope form of the amino acid leucine. We chose ^13^ C_6_-labeled leucine because it is one of the essential amino acids that rapidly crosses the blood brain barrier via facilitative neutral amino acid transport [34]. Intraperitoneal administration of the label was chosen because it is straightforward and quick, and it allows for high bioavailability upon absorption into the bloodstream. Following the injection, we observed a rapid increase in the amount of ^13^ C_6_-leucine in the plasma of the mice over the first hour, as measured by the ratio of labeled to unlabeled free leucine quantified by GC-MS (Figure 1B). After predetermined time points, the mice were euthanized and the brains were quickly removed and frozen. Upon collection of all of the brain samples for each time course, the tissue was then lysed in a 1% Triton X-100 lysis buffer and the protein of interest (apoE and Aβ for this study) was immunoprecipitated with protein-specific antibodies covalently coupled to protein G sepharose beads. Only the cortex of the brain was used in this study; however this technique could easily be applied to measure turnover rates in other regions of the brain. The isolated proteins were eluted off the beads using formic acid, and the concentrated samples were digested with trypsin to generate protein-specific peptides for each protein. These peptide mixtures were then subjected to LC-MS analysis for identification and characterization as described below.

### Mass spectrometry analysis to calculate the ratio of labeled to unlabeled peptide

**Figure 2:**
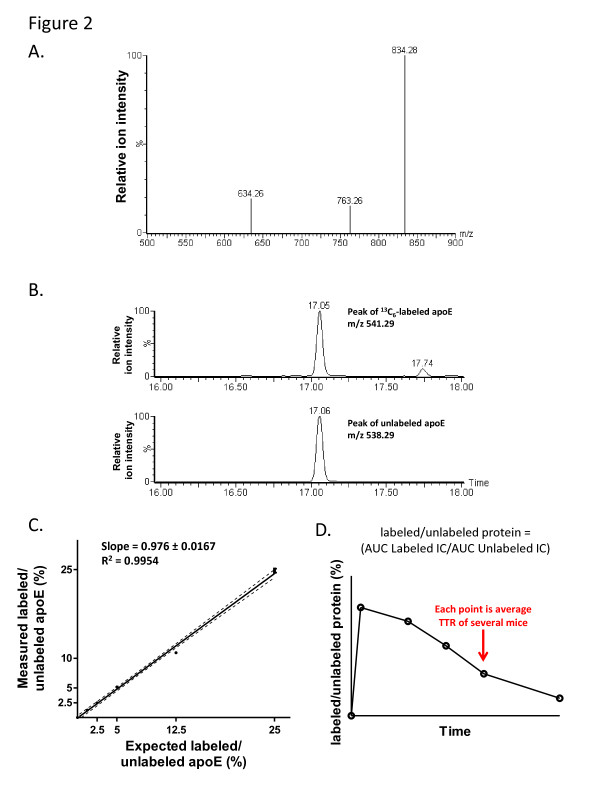
**Tandem mass spectrometry (MS/MS) analysis and quantitation of stable isotope labeled apoE.** (**A**) Tryptic peptides from immunoprecipitated apoE were separated by liquid chromatography and detected using a Xevo TQ-S triple quadrupole mass spectrometer. To facilitate the accurate and specific quantitation of labeled apoE, MRM transitions and conditions were optimized for the parent ion LQAEIFQAR. MS/MS spectrum for the product MRM transitions is shown. A similar analysis was performed for the Aβ specific peptide. (**B**) Representative relative ion count peaks from multiple reaction monitoring (MRM) analysis of the labeled and unlabeled apoE parent peptide LQAEIFQAR are shown [mass charge ratio (m/z) = 541.29 for labeled peptide and 538.29 for unlabeled peptide]. The area under the curve of the MRM ion counts were used for quantitation of the labeled and unlabeled peptide (**C**) Standard curve of labeled apoE. To generate a standard curve for the MS quantitation, primary mouse astrocytes were incubated in culture media with different percentages of labeled leucine. The media of the astrocytes was then collected and the secreted apoE was immunoprecipitated, digested with trypsin, and analyzed using LC-MS. The measured labeled/unlabeled ratios along with the predicted labeled/unlabeled values are shown with a linear regression line (n = 3, dotted lines represent 95% confidence bands). (**D**) Representative diagram of time course of labeled proteins used for kinetic analyses. The labeled/unlabeled ratio was calculated for individual protein samples by dividing the area under the curve of the labeled ion count (IC) by the area under the curve of the unlabeled IC. The labeled/unlabeled ratios were averaged for all of the mouse brain samples collected at each time point. The averaged labeled/unlabeled ratios were then plotted versus time to obtain the kinetic curve for each mouse genotype.

We used a targeted LC-MS approach to accurately and precisely quantify the amount of labeled apoE and Aβ in the brain. Multiple reaction monitoring (MRM) assays were developed for each protein by first selecting a peptide that shows high MS signal intensity, contains only one leucine residue, and is specific for each protein (LQAEIFQAR for apoE, LVFFAEDVGSNK for Aβ). Synthetic peptides were then directly injected into the MS to select and optimize the MRM product transitions for each parent ion (Figure 2A, Table 1, details of the MRM optimization protocol are found in the METHODS section). These parent/precursor ion groupings were then used for the relative quantitation of the labeled and unlabeled peptides from the brain sample. The area under the curve (AUC) of the MRM ion count during the course of the parent ion elution was calculated for both the labeled and unlabeled peptide peaks (Figure 2B). The AUC for the labeled peak was then divided by the AUC for the unlabeled peak to calculate the tracer-to-tracee ratio (TTR) for each sample.

**Table 1:** MRM transitions used for apoE and Aβ analysis

**Protein**	**Peptide sequence**	**Precursor m/z**	**Product m/z**	**Collision Energy (V)**
**ApoE**	LQAEIFQAR	538.2852	634.2609	14
**ApoE**	LQAEIFQAR	538.2852	763.2590	12
**ApoE**	LQAEIFQAR	538.2852	834.2775	14
**ApoE**	[^13^ C_6_]LQAEIFQAR	541.2852	634.2609	14
**ApoE**	[^13^ C_6_]LQAEIFQAR	541.2852	763.2590	12
**ApoE**	[^13^ C_6_]LQAEIFQAR	541.2852	834.2775	14
**Aβ**	LVFFAEDVGSNK	663.3405	819.3840	24
**Aβ**	LVFFAEDVGSNK	663.3405	966.4520	24
**Aβ**	LVFFAEDVGSNK	663.3405	1113.5210	24
**Aβ**	[^13^ C_6_]LVFFAEDVGSNK	666.3500	819.3840	24
**Aβ**	[^13^ C_6_]LVFFAEDVGSNK	666.3500	966.4520	24
**Aβ**	[^13^ C_6_]LVFFAEDVGSNK	666.3500	1113.5210	24

In order to accurately compare the TTR values between individual brain samples and across cohorts of animals, we developed standard curves for both apoE and Aβ using the stable isotope labeling of amino acids in cell culture (SILAC) method [35]. The standard curve for Aβ was generated as previously described [17]. Since astrocytes are the main cell type in the brain that produce apoE [20], we used primary astrocyte cultures to produce a labeled apoE standard curve. To label newly synthesized apoE, the astrocytes were cultured in leucine-free media supplemented with different ratios of ^13^ C_6_-leucine to unlabeled leucine. Under these conditions, all apoE that was synthesized and secreted into the cell media are labeled with the percentage of ^13^ C_6_-leucine provided to the cells. After a 48 hr incubation, the cell media was collected and apoE was immunoprecipitated. Following trypsin digestion, the apoE peptides were subjected to LC-MS as described above. The measured amount of percent labeled apoE gave values that were very close to the expected values (Figure 2C). The linear fit had a slope of 0.976 and an R^2^ value of 0.9954. The apoE and Aβ media standards were used in all subsequent experiments to calibrate the quantitation of the mouse brain samples.

In order to calculate the fractional clearance rates (FCRs) of apoE and Aβ from the brain for each cohort of mice, mice were injected with ^13^ C_6_-leucine and the brains were removed at predetermined time points following the label administration. To determine the optimal time course for analyzing apoE and Aβ clearance, a preliminary experiment was performed with 1 to 2 mice at each time point. For the actual experiments, several mice (n = 5 to 6) were labeled for each time point and the TTR values were averaged. The averaged TTR values were then plotted over time for the whole cohort of mice to obtain the kinetic time course of label disappearance (Figure 2D). The negative of the slope of the monoexponential curve was then calculated in order to determine the fractional clearance rate for each protein from the brain. Through the use of cell-derived labeled protein standards, this technique yielded highly reproducible results. For instance, the labeling of three different cohorts of wild-type (Wt) control mice resulted in similar FCR values for apoE (Table 2; 0.093, 0.10, and 0.09 pools/hr, mean = 0.094 pools/h ± 0.003). These cohorts did not have the same amount of apoE protein as measured by ELISA [Pool size (PS) in Table 2, likely because of the different genetic backgrounds of the mice. Studies have shown that different mouse strains can have up to a four-fold difference in plasma apoE levels [36,37]. Since we observed similar FCR values from the Wt mice across the different genetic strains, our results suggest that the different brain apoE levels are not caused by variations in apoE catabolism.

**Table 2:** Pool Sizes (PS), Fractional Clearance Rates (FCR), Production Rates (PR), and Half-lives for ApoE by Mouse Genotype

**Genotype**	**PS (ng/mg)**	**FCR (pools/hr)**	**PR (ng/mg/hr)**	Half-life (t_1/2_, hrs)
Wt (LDLR Tg control)	125.4 ± 9.1	0.093 ± 0.011	11.69 ± 1.64	7.5
LDLR Tg	44.7 ± 1.9	0.25 ± 0.019	11.07 ± 0.96	2.8
*P*	<0.0001	<0.0001	0.75	
Wt (ABCA1 Tg control)	135.5 ± 4.8	0.10 ± 0.019	14.08 ± 1.64	6.9
ABCA1 Tg	109.2 ± 3.8	0.15 ± 0.022	16.81 ± 0.96	4.6
*P*	<0.0001	0.106	0.46	
Wt (ABCA1 −/− control)	230.5 ± 12.1	0.09 ± 0.015	20.76 ± 3.64	7.7
ABCA1 −/−	117.6 ± 8.0	0.17 ± 0.010	20.41 ± 2.13	4.1
*P*	<0.0001	<0.0001	0.94	

### LDLR overexpression enhances the apoE clearance rate

**Figure 3:**
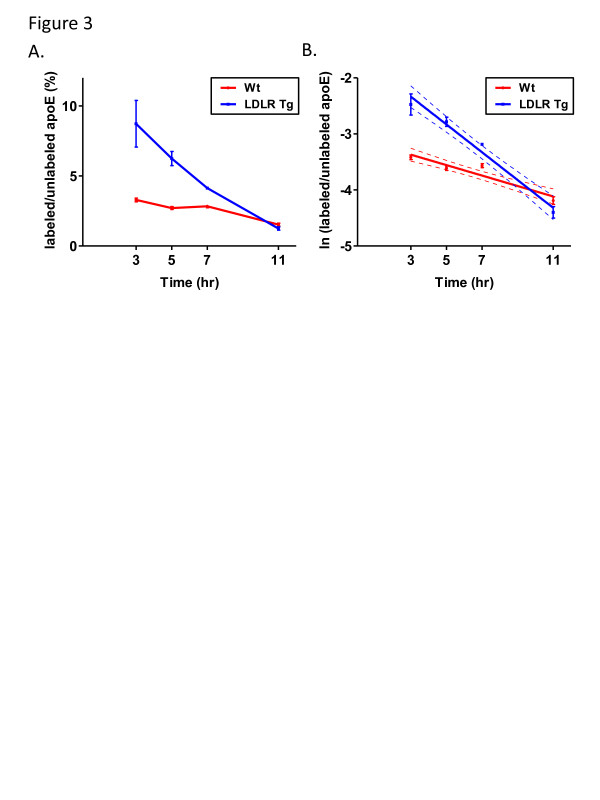
^**13**^ **C**_**6**_**-leucine brain apoE labeling in the presence of increased LDLR levels.** (**A**) Wildtype and LDLR transgenic mice (3.5 months old) were labeled with ^13^ C_6_-leucine and the brains isolated after predetermined time points. ApoE was then immunoprecipitated from the cortex and the labeled/unlabeled ratios calculated via LC-MS. The labeled/unlabeled ratios were then plotted versus time for each genotype. (**B**) To calculate the FCR, the natural log of the labeled/unlabeled ratios were plotted over time and the monoexponential slopes were calculated (n = 5 mice per time point, error bars represent SEM, dotted lines represent 95% confidence band).

To verify that our labeling technique could measure differences in the clearance rates of proteins, we analyzed the effect of overexpressing LDLR on the apoE clearance rate from the brain. LDLR is a receptor that binds to apolipoprotein B and apoE in the periphery to facilitate the uptake of cholesterol-laden lipoproteins by cells [38]. Previously we have shown that LDLR transgenic mice that overexpress LDLR in the brain have significantly decreased levels of brain apoE [27]. We therefore hypothesized that the apoE clearance rate in the brain would be increased in mice that have elevated LDLR levels. Wt and LDLR Tg mice were labeled with ^13^ C_6_-leucine and the apoE TTR values were measured after pre-determined time points. Plots of the TTR values (presented as labeled/unlabeled apoE) over time along with the monoexponential slopes of these curves are shown in Figures 3A and 3B, respectively. Table 2 shows the pool sizes (PS), fractional clearance rates (FCRs), and production rates (PR) for apoE from each genotype. The FCR of apoE was 2.7-fold faster in the LDLR Tg mice in comparison to the Wt mice, while the apoE pool size was 2.8-fold higher for the Wt mice in comparison to the LDLR Tg mice. These values were used to estimate the PR values for both genotypes (see METHODS section for explanation of PR calculation), and no statistical difference in the PR was observed between Wt and LDLR Tg mice. These results convincingly demonstrate that apoE clearance is enhanced in the brains of LDLR Tg mice, providing the likely explanation for the decreased total apoE protein levels. We therefore concluded that the pulsed ^13^ C_6_-leucine injection labeling technique is effective for measuring the clearance of proteins from the brain, and could be used to detect differences in FCRs between genetically modified mouse models.

### Effect of ABCA1 levels on apoE and Aβ clearance rates

ABCA1 is a transmembrane protein that plays an important role in the efflux of cholesterol and phospholipids to lipid-poor apolipoproteins [39]. In the brain, the level of ABCA1 has been shown to modulate the extent of apoE lipidation and apoE levels. Surprisingly, both deletion and overexpression of ABCA1 in the mouse brain led to a decrease in apoE levels [28-30]. However, the apoE containing lipoprotein particles isolated from the cerebral spinal fluid (CSF) of ABCA1^−/−^ mice were poorly lipidated, while those from the CSF of mice overexpressing ABCA1 had higher levels of lipidation compared to Wt animals [28,30]. Because lowering apoE levels decreased amyloid deposition in the mouse brain[25], it was hypothesized that altering ABCA1 levels would also alter amyloid deposition in APP transgenic mice. Despite decreased apoE levels with both ABCA1 deletion and overexpression, only ABCA1 overexpression caused a significant decrease in amyloid load in the mouse brain of APP transgenic animals [30]. The amyloid load in APP transgenic mice deficient in ABCA1 did not change, or even increased, when ABCA1^−/−^ mice were crossed to various APP transgenic models [40-42]. Because of the opposing effects of ABCA1 deletion and overexpression on Aβ accumulation, ABCA1 likely alters Aβ levels through a mechanism distinct from modulating apoE levels. One proposed mechanism is ABCA1 levels could alter Aβ clearance from the brain [30,43]; however this hypothesis has never been tested *in vivo*. Therefore, we used our stable isotope labeling technique to study the effect of ABCA1 levels on both apoE and Aβ clearance rates in the mouse brain.

**Figure 4:**
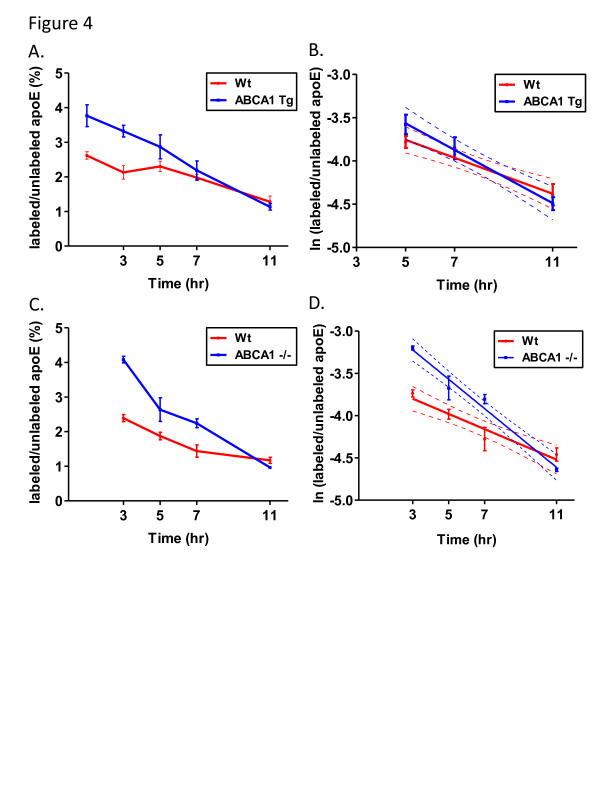
^**13**^**C**_**6**_**-leucine brain apoE labeling in the presence of ABCA1 overexpression and deletion.** (**A**) Cohorts of wildtype and ABCA1 transgenic mice and (**C**) wildtype and ABCA1^−/−^ mice were labeled with ^13^ C_6_-leucine and the brains isolated after predetermined time points. Note that separate groups of animals were used as the wildtype controls for the ABCA1 Tg and ABCA1^−/−^ mice. The apoE labeled/unlabeled ratios were then calculated and the data plotted as in Figure 3. For FCR measurements, the monoexponential slopes were measured for (**B**) ABCA1 Tg and (**D**) ABCA1^−/−^ mice and their respective Wt controls (n = 5-6 mice per time point, error bars represent SEM, dotted lines represent 95% confidence band).

**Figure 5:**
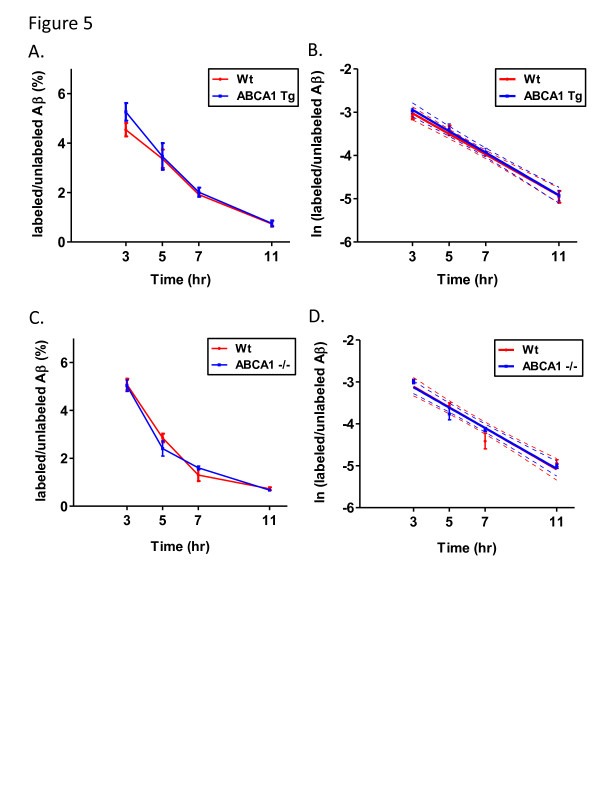
^**13**^**C**_**6**_**-leucine brain Aβ labeling in the presence of ABCA1 overexpression and deletion.** (**A**) Cohorts of wildtype and ABCA1 transgenic mice and (**C**) wildtype and ABCA1^−/−^ mice were labeled with ^13^ C_6_-leucine and the brains isolated after predetermined time points. Total Aβ was immunoprecipitated from the cortex and the labeled/unlabeled ratios were then plotted versus time for each genotype. For FCR measurements, the data was plotted as in 3B and the monoexponential slopes were calculated for both (**B**) ABCA1 Tg and (**D**) ABCA1^−/−^ mice and their respective Wt controls (n = 5-6 mice per time point, error bars represent SEM, dotted lines represent 95% confidence band).

To generate APP transgenic mice that either overexpressed or were deficient in ABCA1 levels, we crossed PDAPP mice with ABCA1 Tg and ABCA1^−/−^ mice. These animals were then injected with ^13^ C_6_-leucine and the FCRs of both apoE and Aβ were measured as described above for the LDLR Tg animals. To limit complications due to Aβ extraction from tissue with amyloid plaques, all experiments were performed on young animals (3.5 months old) prior to the onset of detectable plaque deposition. Plots of the labeled/unlabeled protein values over time along with the monoexponential slopes of these curves are shown in Figures 4 and 5 for apoE and Aβ, respectively. The PS, FCR, and PR values for apoE and Aβ are given in Table 2 and Table 3, respectively. The apoE FCR was 1.5-fold faster in ABCA1 Tg mice and 1.9-fold faster in ABCA1^−/−^ mice compared to Wt mice; however the difference was only significant for the ABCA1^−/−^ mice. The apoE PS decreased by 20% in ABCA1 Tg mice and by 51% in ABCA1^−/−^ mice compared to Wt mice. No differences were observed in the PR of apoE. For Aβ, no differences were observed in the FCR, PS, or PR values (Table 3).

**Table 3:** Pool Sizes (PS), Fractional Clearance Rates (FCR), Production Rates (PR), and Half-lives for Aβ by Mouse Genotype

**Genotype**	**PS (pg/mg)**	**FCR (pools/hr)**	**PR (pg/mg/hr)**	**Half-life** **(t**_**1/2**_**, hrs)**
Wt (ABCA1 Tg control)	11.23 ± 0.56	0.238 ± 0.016	2.65 ± 0.21	2.91
ABCA1 Tg	11.14 ± 0.46	0.247 ± 0.016	2.77 ± 0.23	2.81
*P*	0.90	0.69	0.68	
Wt (ABCA1 −/− control)	7.67 ± 0.26	0.247 ± 0.023	1.91 ± 0.15	2.81
ABCA1 −/−	7.87 ± 0.33	0.243 ± 0.016	1.89 ± 0.19	2.85
*P*	0.64	0.88	0.94	

These results demonstrate that both ABCA1 overexpression and deletion increase the fractional clearance rate of apoE from the brain, but have no effect on the Aβ fractional clearance rate. In terms of apoE, the results from the ABCA1^−/−^ mice parallel nicely with plasma kinetic studies performed in humans with loss-of-function mutations in ABCA1. These studies demonstrated that the catabolism of both high-density lipoprotein (HDL) and low-density lipoprotein apolipoprotein B-100 (LDL B-100) were increased in the plasma of these individuals [44,45], suggesting decreased stability of poorly lipidated lipoprotein particles. The lipidation of apoE also significantly alters its propensity to bind to LDLR, with increased lipid levels leading to enhanced binding [46]. As a result, the faster apoE clearance rate in the ABCA1 Tg mice may be due to increased LDLR-mediated clearance of the more highly lipidated apoE-containing lipoprotein particles. Therefore, though both ABCA1 overexpression and deletion led to enhanced apoE clearance, the mechanism underlying the difference in each case may be different.

In regards to Aβ, our results suggest that at a young age prior to the onset of plaque deposition, ABCA1 levels do not alter Aβ clearance from the mouse brain. A previous study measuring the disappearance of radiolabeled Aβ injected into the brains of ABCA1^−/−^ mice also found no effect on Aβ clearance across the blood–brain barrier [47]. Though we cannot rule out the possibility that changes in Aβ clearance develop as the mice age, our current data suggests that the effect of ABCA1 on Aβ deposition in the brain may not occur due to altered metabolism of Aβ. Rather, it is possible that the effect of ABCA1 relates to altered modulation of differentially lipidated forms of apoE on Aβ aggregation or fibrillogenesis.

## Conclusions

In this study, we describe a stable isotope pulse labeling kinetics (SILK) technique that can be used to measure the clearance of proteins from the mouse brain. The non-radioactive labeling method is safe, straightforward, and does not require administration of the stable isotope via the diet. Label administration to the mice is consistent and easily controlled by altering the amount injected. Since the stable isotope quickly appears in both plasma and brain within minutes of the injection, this technique is particularly suitable for measuring the kinetics of proteins that turn over rapidly. The SILK technique is not particularly expensive and can be applied in any laboratory setting that has access to MS instrumentation. The primary costs associated with this technique are the purchase of the stable isotope and the generation and maintenance of the mice prior to injection. In terms of time, the labeling of the mice and collection of tissue is the most laborious aspect of this technique. Once the brain tissue is collected for the whole cohort of mice, preparation and processing of the samples for MS analysis is extremely efficient because all of the samples can be processed in parallel. It takes about one week to complete the labeling of mice and preparation of samples for MS analysis.

We demonstrate that this labeling technique is particularly useful for comparing the kinetics of a protein in cohorts of mice with different genetic manipulations. To show the applicability of this technique to test a hypothesis pertinent to neurodegenerative disease, the effect of ABCA1 levels on the clearance of Aβ from the mouse brain were measured *in vivo* for the first time. Increasing ABCA1 or deleting ABCA1 from the brain had no effect on Aβ clearance. Consequently, ABCA1 likely regulates Aβ deposition in the brain through a mechanism other than altering Aβ metabolism, such as through modulating the propensity of Aβ to aggregate. Previous studies have measured Aβ clearance from the brain either using ^35^ S]methionine labeling [48], or by measuring the disappearance of Aβ following the pharmacological inhibition of Aβ production [49-51]. The half-life of Aβ clearance in these studies ranged from 30 min to 2 h. Consistent with these studies, we observed a half-life for Aβ of approximately 2.8-2.9 h (Table 3). In comparison to other studies, our technique has unique advantages in that it does not require a radioactive tracer and kinetics are determined in the steady-state, which does not occur with the inhibition of Aβ production.

We propose that this method of stable isotope labeling, and its applicability to studying the clearance of proteins in genetically modified mouse models, will be useful in studying the kinetics of proteins implicated in other neurodegenerative diseases, such as synuclein, tau, and huntingtin. We also hope that this technique will aid the development and characterization of novel therapeutics that target protein metabolism in neurodegeneration.

## Methods

### Materials

^13^ C_6_-leucine was obtained from Cambridge Isotope Laboratories (Andover, MA, USA). HJ5.2 (Aβ) and HJ6.3 (ApoE) antibodies were made in-house. Protein G Sepharose 4 Fast Flow beads were obtained from GE Healthcare (Piscataway, NJ, USA). Formic acid (Optima LC-MS) was obtained from Fisher Scientific and triethylammonium bicarbonate was obtained from Sigma-Aldrich (St. Louis, MO, USA). Trypsin Gold (mass spec grade) was purchased from Promega (Madison, WI, USA).

### Animal labeling and tissue collection

The production and characterization of the LDLR transgenic and ABCA1 transgenic mice have been previously described [27,30]. ABCA1^+/−^ mice on a DBA background were obtained from the Jackson Laboratory (Bar Harbor, ME, USA). PDAPP mice on a C57/BL/6J background were a generous gift from Eli Lilly (Indianapolis, IN, USA). LDLR Tg^+/−^ mice were bred to Wt mice to generate mice that were LDLR Tg^+/−^ and LDLR Tg^−/−^. ABCA1 Tg mice were backcrossed to C57/BL/6J mice for 8 generations, and then crossed to DBA mice. PDAPP mice were also crossed to DBA mice and ABCA1^+/−^ mice were crossed to C57/BL/6J mice to create strains that were on a 50% C57/BL/6J/50%DBA background. The ABCA1 Tg^+/−^ and PDAPP^+/−^ mice were then bred to each other to generate ABCA1 Tg^+/−^/PDAPP^+/−^ and ABCA1 Tg^−/−^/PDAPP^+/−^ mice that were used for the experiments. ABCA1^+/−^ were crossed to PDAPP^+/−^ mice to generate mice that were PDAPP^+/−^/ABCA1^+/−^. These mice were then bred to ABCA1^+/−^ mice to generate mice that were PDAPP^+/−^/ABCA1^+/+^, PDAPP^+/−^/ABCA1^+/−^, and PDAPP^+/−^/ABCA1^−/−^. The PDAPP^+/−^/ABCA1^+/+^ and PDAPP^+/−^/ABCA1^−/−^ mice were used for all experiments. Mice were maintained under constant light/dark conditions and had free access to food and water. All experimental protocols were approved by the Animal Studies Committee at Washington University in St. Louis.

Prior to injection, the ^13^ C_6_-leucine was dissolved in medical-grade normal saline to a concentration of 7.5 mg/mL. The mice were weighed and then intraperitoneally injected with the ^13^ C_6_-leucine (200 mg/kg of body weight). After predetermined time points, the animals were anesthetized and the blood was collected by cardiac puncture. The mice were then perfused with PBS-heparin and regional brain dissection was performed. All brain samples were subsequently frozen on dry ice.

### Primary astrocyte cell culture and *in vitro labeling*

Primary astrocytes were cultured from postnatal day (P1) C57/BL/6J mouse pups as described previously [27]. Cells were cultured in serum-containing growth media (DME/F12, 15% fetal bovine serum, 10 ng/mL epidermal growth factor, 100 units/mL penicillin/streptomycin, and 1 mM sodium pyruvate) until they reached 70% confluency. The cell media was then changed to serum free media that did not contain any leucine (DME/F12 without leucine prepared by the Washington University Tissue Culture Support Center, N2 growth supplement, 100 units/mL penicillin/streptomycin, and 1 mM sodium pyruvate) and cultured for 12 h. ^13^ C_6_-leucine was then diluted into unlabeled leucine to make labeled/unlabeled percentages that were either 0, 1.25, 2.5, 5, 10, or 20%. These different percent-labeled leucine solutions were then added to separate flasks of primary astrocytes, and the cells were cultured for an additional 48 h. The media was then collected from the cells, spun down at 1500 rpm to clear cellular debris, and stored at −80°C.

### ApoE and Aβ immunoprecipitation

Antibody beads were prepared by covalently binding either HJ6.3 (apoE) or HJ5.2 (Aβ) to Protein G Sepharose 4 Fast Flow beads. The beads initially were washed 3 times with ice-cold PBS and then resuspended in ice-cold PBS to make a 50% slurry of beads. 300 μL of the washed 50% beads were then mixed with antibody (0.4 μg/μL of 50% bead mixture), 10 μL of 1% Triton X-100 and ice-cold PBS to make a final volume of 1000 μL. This mixture was then tumble incubated overnight at 4°C. The beads were then washed 3 times with 1% Triton X-100 lysis buffer (Triton X-100, 150 mM NaCl, 50 mM Tris–HCl) and 2 times with 0.2 M triethanolamine (pH = 8.2). Freshly prepared dimethyl pimelimidate in 0.2 M triethanolamine (pH = 8.2) was then added to the beads, followed by a 30 min incubation with tumbling at room temperature to allow for crosslinking. The beads were then washed once with 50 mM Tris (pH = 7.5) to stop the crosslinking reaction, and twice with 0.1% Triton X-100 in PBS. The washing solution was removed by vacuum aspiration, and the beads were resuspended in PBS to make a 50% bead slurry.

Brain cortex samples were weighed and 1% Triton X-100 lysis buffer (Triton X-100, 150 mM NaCl, 50 mM Tris–HCl, 1 X Roche Complete Protease Tablet) was added at a concentration of 150 mg brain tissue/μL of lysis buffer. The samples were then sonicated (2 rounds of 20 1-sec pulses) and centrifuged at 14,000 rpm for 30 min. The supernatant was collected and used for subsequent immunoprecipitation steps. Brain lysates and cell media were pre-cleared with beads not conjugated to antibody by tumble incubating the samples with 50 μL of the 50% bead slurry for 4 h at 4°C. The pre-cleared lysate and media samples were then tumble incubated with antibody-conjugated beads overnight at 4°C. The beads were then washed 3 times with PBS and 3 times with 25 mM triethylammonium bicarbonate (TEABC). Following the last TEABC wash, the TEABC was removed via vacuum aspiration with a pipette tip. Formic acid was then added to the beads to elute the bound proteins, and the mixture was vortexed for 20 min. The beads were then centrifuged at 14,000 rpm for 5 min and the supernatant was collected from the beads. The formic acid supernatant was transferred to a new microcentrifuge tube and evaporated in a Savant SpeedVac for 60 min (37°C). The dried proteins were then resuspended in 20% acetonitrile/80% 25 mM TEABC and vortexed for 30 min. The samples were then digested with 500 ng of mass spectrometry-grade trypsin (Promega) and incubated at 37°C for 16 h. The digested samples were dried again by vacuum evaporation, resuspended in 10% acetonitrile and 0.1% formic acid in water, and transferred to mass spec vials.

### Liquid chromatography/mass spectrometry

LC-MS/MS measurements were performed on a Waters Xevo TQ-S triple quadrupole mass spectrometer (Waters Inc., Milford, MA) coupled to a Waters nano-ACQUITY ultra performance liquid chromatography (UPLC) system, equipped with a Waters nano-ESI ionization source. To identify multiple reaction monitoring (MRM) transitions, the synthetic apoE peptide LQAEIFQAR and synthetic Aβ peptide LVFFAEDVGSNK were purchased from AnaSpec, Inc. (Fremont, CA), and directly infused into the LC-MS for automatic tuning of optimized MRM transitions produced by the peptide. For both the apoE and Aβ peptide, optimal conditions were identified as a capillary voltage of 3.3 kV, source temperature of 80°C, cone voltage of 52 V, purge gas flow rate set at 100 L/hr, and cone gas at 50 L/hr. Obtained MRM transitions (Table 1) were then validated by the analysis of apoE and Aβ cell culture media standards. For the actual experiments, all digested peptide samples were kept at 4°C and 1 μL aliquots were injected onto a Waters BEH130 nanoAcquity UPLC column (C18 particle, 1.7 μm, 100 μm × 100 mm). The peptide mixtures were separated on a reverse-phase nanoUPLC operated at a flow rate of 500 nL/min with a gradient mixture of solvents A (0.1% formic acid in water) and B (0.1% formic acid in acetonitrile). For apoE, the column was initially kept at 99% solvent A for 1.5 min, followed by a separation gradient of 1% to 97% solvent B from 1.5 to 18 min. The column was then kept at 97% solvent B for another 5 min followed by 1% solvent B to re-equilibrate for 10 min to prepare for the next injection. For Aβ, the column was initially kept at 90% solvent A for 7.0 min, followed by a separation gradient of 10% to 45% solvent B from 7 to 12 min. The column was then kept at 45% to 95% solvent B from 12 to 14 min, and at 95% solvent B for another 3 min followed by 10% solvent B to re-equilibrate for 15 min to prepare for the next injection. All raw data were acquired and quantified using Waters MassLynx 4.1 software suite. The labeled/unlabeled ratio was obtained by dividing the area under the curve (AUC) of the MRM total ion for the labeled peptide by the AUC for the unlabeled peptide, and converted to tracer-to-tracee ratios (TTRs) by reference to the standard curve.

### Gas chromatography/mass spectrometry

The free leucine tracer-to-tracee ratio was measured from the mouse plasma using GC/MS. Plasma proteins were precipitated with ice-cold acetone, and lipids were extracted using hexane solvent. The resulting aqueous fraction was then dried with a vacuum (Savant Instruments, Farmingdale, NY) and converted to t-butyldimethylsilyl derivatives. The free leucine TTR was then measured by monitoring ions with m/z ratios of 200 (unlabeled) and 203 (labeled) [52].

### Kinetic analysis

The mice were in steady-state conditions, since the amount of apoE and Aβ did not significantly change over the time period of the kinetic analysis. This was determined by measuring the protein level (via ELISA as described below) for the cohorts of mice at each time point following the stable isotope injection, and comparing across groups. At metabolic steady state, the fraction of the pool that is synthesized per unit time equals the fraction of the pool catabolized per unit time (FCR), which can be calculated as the negative of the slope of the natural log of TTR plotted over time [53]. Production rates (PRs) were determined as: PR (protein amount/mg/hr) = [FCR (pools/hr) × protein concentration (protein amount/mL) × lysate volume (mL)]/brain weight (mg). The half-lives (t_½_) were calculated using the equation t_½_ = ln 2/FCR. Protein concentrations of apoE and Aβ in the lysates were determined by protein-specific sandwich ELISAs using in-house antibodies. For apoE, HJ6.2 was used as the coating antibody and biotinylated HJ6.3 as the detection antibody. Pooled C57/BL/6 J mouse plasma was used as a standard. For Aβ, HJ2 (anti-Aβ35-40) and biotinylated HJ5.1 (anti-Aβ13-28) were used as the coating and detection antibody, respectively.

### Statistical analysis

Data were analyzed using GraphPad Prism Software and presented as mean ± standard error of the mean (SEM). For analyzing differences in protein levels and production rates, a two-tailed student’s *t*-test was used. Differences in the FCR values were compared using analysis of covariance (ANCOVA) of the negative of the slope of the natural log of TTR plotted over time, which was determined using linear regression analysis.

## Misc

Jacob M Basak and Jungsu Kim are contributed equally to this work

## Competing interests

DMH and RJB co-founded and are on the scientific advisory board of C2N Diagnostics. BWP provides consultation services for tracer turnover kinetics for C2N Diagnostics.

## Authors’ contributions

JMB, JK, and DMH conceived and designed the experiments. JMB, JK, HJ, and MP labeled mice and collected mouse tissue. JMB and HJ performed all MS sample preparation. YP, KRW, and RJB performed and optimized MS data collection and analysis. BWP assisted with kinetic analysis and interpretation. JMB, JK, BWP, and DMH wrote the paper. All authors revised the manuscript for important intellectual content and gave final approval of the version to be published.
